# Brain metastases as first manifestation of advanced cancer: exploratory analysis of 459 patients at a tertiary care center

**DOI:** 10.1007/s10585-018-9947-1

**Published:** 2018-11-12

**Authors:** L. M. Füreder, G. Widhalm, B. Gatterbauer, K. Dieckmann, J. A. Hainfellner, R. Bartsch, C. C. Zielinski, M. Preusser, A. S. Berghoff

**Affiliations:** 1 Clinical Division of Oncology, Comprehensive Cancer Center CNS Tumors Unit, Department of Medicine I, Medical University of Vienna, Waehringer Guertel 18-20, 1090 Vienna, Austria; 2 Comprehensive Cancer Center, Medical University of Vienna, Vienna, Austria; 3 Department of Neurosurgery, Medical University of Vienna, Vienna, Austria; 4 Department of Radiotherapy, Medical University of Vienna, Vienna, Austria; 5 Institute of Neurology, Medical University of Vienna, Vienna, Austria

**Keywords:** Cancer of unknown primary, Brain metastases, Survival prognosis, Targeted therapies, Graded prognostic assessment

## Abstract

Symptomatic brain metastases (BM) are a frequent and late complication in cancer patients. However, a subgroup of cancer patients presents with BM as the first symptom of metastatic cancer. Here we aimed to analyze the clinical course and prognostic factors of this particular BM patient population. Patients presenting with newly diagnosed BM without a history of metastatic cancer were identified from the Vienna Brain Metastasis Registry. Clinical characteristics and overall survival were retrieved by chart review. 459/2419 (19.0%) BM patients presented with BM as first symptom of advanced cancer. In 374/459 (81.5%) patients, an extracranial primary tumor, most commonly lung cancer, could be identified within 3 months after BM diagnosis. In 85/459 (18.5%) patients no extracranial primary tumor could be identified despite comprehensive diagnostic workup within the first 3 months after diagnosis of BM. Survival of patients with identified extracranial tumor differed only numerically from patients with cancer of unknown primary (CUP), however patients receiving targeted therapy after molecular workup showed significantly enhanced survival (20 months vs. 7 months; p = 0.003; log rank test). The GPA score showed a statistically significant association with median overall survival times in the CUP BM patients (class I: 46 months; class II: 7 months; class III: 4 months; class IV: 2 months; p < 0.001; log rank test). The GPA score has a strong prognostic value in patients with CUP BM and may be useful for patient stratification in the clinical setting. Comprehensive diagnostic workup including advanced imaging techniques and molecular tissue analyses appears to benefit patients by directing specific molecular targeted therapies.

**Electronic supplementary material:**

The online version of this article (10.1007/s10585-018-9947-1) contains supplementary material, which is available to authorized users.

## Introduction

Brain metastases (BM) are a frequent and devastating complication occurring in up to 10–30% of patients with advanced cancer. Most patients experience BM rather late during their clinical course. However, a fraction of patients has been reported to present with BM as first symptom of cancer [1–3]. The clinical management of patients with simultaneous BM and extracranial advanced cancer includes the local therapy to control BM symptoms and comprehensive diagnostic workup to guide further treatment strategies. Identification of the primary tumor is possible in the majority of patients with lung cancer being the most frequent extracranial primary diagnosis. The remaining patients are left with the diagnosis of cancer of unknown primary (CUP) and the resulting challenges in further treatment. Only limited information on the clinical course, survival outcomes and prognostic factors of CUP BM patients are available, although such data would be of interest for the clinical management and planning of clinical trials in this particular patient cohort with high medical need. Therefore, we aimed to analyze clinical characteristics and prognostic factors in patients presenting with BM as first manifestation of metastatic cancer and in particular the sub-cohort of CUP BM patients.

## Methods

### Patients

Patients with newly diagnosed BM without a history of metastatic cancer referred to the Medical University of Vienna between 1990 and 2015 were identified from the Vienna Brain Metastasis Registry. All included patients presented with new-onset neurological symptoms in absence of any extracranial symptoms or a history of metastatic cancer. Patients with previous history of cancer were only included if the previous diagnosis was locally restricted cancer treated with curative intent at least 5 years before the occurrence of neurological symptoms.

**Fig. 1:**
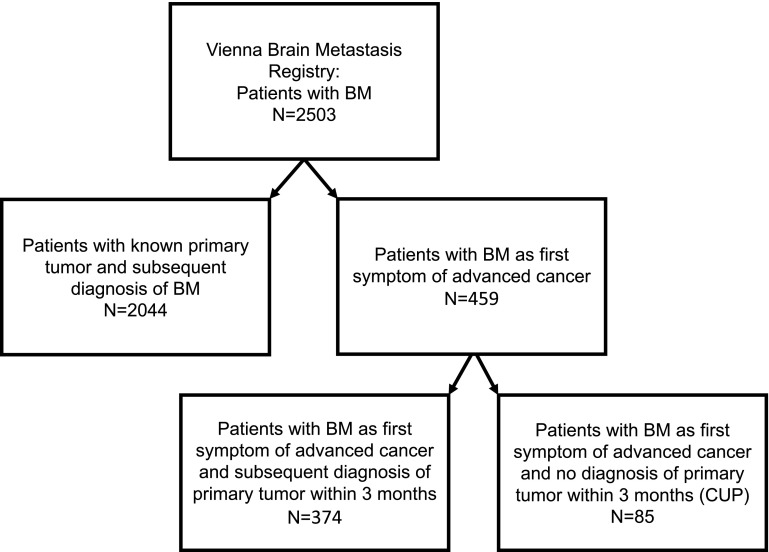
Consort diagram displaying the included patient populations

Patients were diagnosed as CUP if metastatic cancer was histologically confirmed but medical history, physical examination, basic biochemistry battery, full blood count, urinalysis, stool occult blood testing, immunohistochemistry with specific markers, imaging technology with chest X-ray, computer tomography (CT) of the thorax, abdomen and pelvis, as well as mammography, magnetic resonance imaging (MRI) and positron emission tomography (PET) scan in certain cases, failed to identify a specific underlying primary tumor [1, 4]. The first set of these intensive investigations was completed after at the maximum of 3 months and treatment decision were set. Therefore, we defined CUP as patients in whom the underlying tumor entity could not be identified even after intensive diagnostic assessment and complete staging within 3 months after BM diagnosis. Figure 1 gives an overview of the included and analyzed patient cohort. Clinical data on patient characteristics, diagnostic procedures on primary tumor identification and the clinical course of disease were retrieved by chart review. Neurological symptoms, signs of increased cranial pressure and epileptic seizure were recorded and patients with 2 or more symptoms were defined as highly symptomatic. An extracranial tumorous/metastatic lesion was defined as a lesion located in an organ other than the brain but not matching as a primary location to the obtained histology of the BM. Further, extracranial tumorous/metastatic lesion was defined as an extracranial lesion presenting in the histological work-up not with characteristic histological features of malignancies of this particular organ and therefore rather presenting a metastasis than a primary tumor. The Graded Prognostic Assessment (GPA) was calculated as previously published based on age, Karnofsky Performance Score (KPS), status of extracranial disease and number of BM [5–7]. Survival data were acquired from the database of National Cancer Registry of Austria and the Austrian Brain Tumor Registry. The study was conducted under approval of the ethics committee of the Medical University of Vienna (078/2004).

### Statistical analysis

Differences between groups were assessed using the Chi square test, the Kruskal–Wallis test and the Mann–Whitney U test as appropriate. Overall survival (OS) time from BM was measured in months and defined as interval from radiological detection of BM to death or last follow-up. The Kaplan–Meier product limit method was used to estimate OS. To estimate OS differences between groups the log-rank test was used. To estimate the prognostic impact of latency from BM diagnosis to primary tumor diagnosis the Cox-regression model along with a time-dependent covariate was used. Clinical data of patients presenting with BM as first symptom of cancer was compared to patients with known primary tumor available from a previous publication [1, 8]. A two-tailed p-value ≤ 0.05 was considered to indicate statistical significance. Statistical analysis was performed with Statistical Package for the Social Sciences (SPSS®) 23.0 software (SPSS Inc., Chicago, IL, USA).

## Results

### Patient characteristics

459/2419 (19.0%) patients treated for newly diagnosed BM between 1990 and 2015 at the Medical University of Vienna presented with BM without any history of extracranial metastatic cancer and were therefore included for further analysis (Fig. 1). All patients were referred due to new onset of neurological symptoms (184/459 (40.1%) headaches, 416/459 (90.6%) neurological deficits, 206/459 (44.9%) signs of increased intracranial pressure, 91/459 (19.8%) seizures). Table 1 lists further patients’ characteristics.

**Table 1:** Clinical characteristics of patients with brain metastases as first manifestation of cancer

Characteristic	Entire population (n = 459)	
	n	%
Median age at diagnosis of BM, years (range)	59 (27–88)	
Gender		
Female	191	41.6
Male	268	58.4
Primary tumor type		
Lung cancer	308	67.1
Breast cancer	4	0.9
Melanoma	26	5.7
Primary lesion occult	23	88.5
Primary lesion detected	3	11.5
Renal cell carcinoma	21	4.6
Colorectal carcinoma	9	2.0
Cancer of unknown primary (CUP)	85	18.5
Primary not detected	60	70.6
Primary detected after > 3 months diagnostic investigation	15	17.6
Primary detected at autopsy	10	11.7
Others	6	1.3
Symptoms		
Headaches	184	40.1
Neurological deficits	416	90.6
Signs of increased intracranial pressure	206	44.9
Seizures	91	19.8
Median KPS at BM diagnosis (range)	80 (10–100)	
Number of BM		
1	216	47.1
2–3	133	29.0
> 3	110	24.0
Extracranial tumorous lesions		
No	176	38.3
Yes	283	61.7
Pulmonary tumorous lesions		
Yes	81	17.6
No	378	82.4
Liver tumorous lesions		
Yes	37	8.1
No	422	91.9
1st line therapy for newly diagnosed BM		
Surgery	276	60.1
Gamma Knife	94	20.5
WBRT	79	17.2
Best supportive care	8	1.7
Chemotherapy	2	0.4
Chemotherapy after diagnosis of BM		
Yes	222	48.4
No	237	51.6
Targeted therapy after diagnosis of BM		
Yes	29	6.3
No	430	93.7
Type of targeted therapy		
EGFR tyrosine kinase inhibitor	16	3.5
Anti-VEGF therapy	6	1.3
Anti-HER2 therapy	1	0.2
Immune modulating therapy	4	0.9
BRAF inhibitor	2	0.4
Targetable mutation		
EGFR mutation	4	0.9
HER2 overexpression	1	0.2
BRAF mutation	2	0.4
GPA		
Class I	51	11.1
Class II	61	13.3
Class III	276	60.1
Class IV	71	15.5
Extracranial progression after 1st line therapy		
Yes	206	44.9
No	253	55.1
Intracranial progression after 1st line therapy		
Yes	186	40.5
No	273	59.5
Median OS from diagnosis of BM (range)	8 (0–230)	

20/459 (4.4%) patients had been treated for localized cancer in curative intend at least 5 years prior to diagnosis of BM (2/459 (0.4%) breast cancer; 4/459 (0.9%) melanoma; 1/459 (0.2%) renal cell carcinoma; 1/459 (0.2%) colorectal carcinoma; 3/459 (0.7%) prostate cancer; 4/459 (0.9%) cervical cancer; 1/459 (0.2%) vaginal cancer; 2/459 (0.4%) bladder cancer; 1/459 (0.2%) squamous cell skin cancer; 1/459 (0.2%) renal cell carcinoma, prostate cancer and non-Hodgkin lymphoma). In all of these 20 cases, another primary tumor causing the BM was identified at diagnostic workup (Supplemental Table 1).

### Clinical characteristics of patients presenting with brain metastasis as first manifestation of advanced cancer

Compared to patients with known primary tumor in the Vienna Brain Metastasis Registry (n = 2044; Fig. 1), patients with BM as first symptom of cancer (n = 459) more frequently presented with at least one BM with a diameter of 3 cm or larger (50.1% vs. 30.5%; p < 0.001; Chi Square test). Patients with BM as first sign of cancer were more likely to suffer from highly symptomatic intracranial disease compared to patients with known primary tumor (52.9% vs. 32.0%; p < 0.001; Chi Square test). No difference in number of BM was observed between BM patients with known primary tumor and patients presenting with BM as first manifestation of advanced cancer (1: 47.1% vs. 48.4%; 2–3: 29.0%–28.1%; > 3: 24.0% vs. 23.5%; p = 0.866; Chi Square test). No difference in BM location was evident between patients with known primary tumor and patients with BM as first symptom of advanced cancer as the proportion of patients with supratentorial (61.1% vs. 61.9%), infratentorial (13.0% vs. 13.1%) and bilateral BM (24.9% vs. 24.8%) were comparable (p = 0.770; Chi Square test) among both groups. More detailed, cerebellar BM (24.8% vs. 24.8%; p = 0.971; Chi Square test), frontal BM (36.8% vs. 33.6%; p = 0.184; Chi Square test), occipital BM (17.4% vs. 23.0%; p = 0.510; Chi Square test) and temporal BM (18.3% vs. 19.3%; p = 0.631; Chi Square test) presented at comparable proportions among the two groups. Only BM in parietal localization were statistically significantly more frequently observed in patients with BM as first symptom of advanced cancer (29.6%) compared to patients with known primary tumor (23.0%; p = 0.029; Chi Square test).

Extracranial tumorous lesions were statistically significantly more frequently observed in patients with known primary tumor (1428/2044; 69.9%) compared to patients with BM as first symptom of cancer (175/459; 38.1%; p < 0.001; Chi Square test). In univariate analysis, patients with known primary tumor presented with a median survival time of 6 months after diagnosis of BM compared to 8 months in patients with BM as first symptom of advanced cancer (p = 0.003; log rank test). However, multivariate analysis that included the presence of extracranial tumorous lesions (HR 0.7; 95% CI 0.679–0.808; p < 0.001; cox regression model) and known primary tumor status (HR 1.1; 95% CI 0.955–1.185; p = 0.261; cox regression model), did not show statically significant association of known primary tumor status and survival prognosis. 170/2044 (8.3%) patients with known primary tumor received a targeted therapy as first-line systemic treatment approach after the local BM therapy. Importantly, patients receiving a targeted therapy presented with an improved survival prognosis compared to patients without targeted therapy (6 months vs. 11 months; p > 0.001; log rank test).

### Diagnostic procedures in patients presenting with brain metastasis as first manifestation of advanced cancer

Cranial imaging with CT (341/459 (74.3%)) or MRT (111/459 (24.2%)) was performed in all patients and revealed the diagnosis of an intracranial tumor mass at initial presentation. Based on radiological findings, BM biopsy or resection to obtain tissue for histological investigation was performed in 302/459 (65.8%) patients. In 212/459 (46.2%) patients primary tumor tissue could be obtained for diagnostic workup. In these patients, CT scan (193/459, 42.0%) followed by chest X-ray (101/459; 22.0%) and FDG-PET (15/459; 3.3%) most frequently guided the diagnostic approach to obtain extracranial tumor material (Table 2). In 159/459 (34.6%) patients the analysis of the extracranial tissue confirmed the suspected diagnosis, while in 53/459 (11.5%) patients molecular analysis of the extracranial tumor tissue did not lead to any definitive, histological diagnosis. In 33/459 (7.2%) patients a histological diagnosis was made based on BM histology only as no extracranial primary tumor tissue could be obtained. Detailed information on diagnostic workup is presented in Table 2.

**Table 2:** Diagnostic assessment

Characteristic	Entire population (n = 459)	
	n	%
Median latency to primary detection, days (range)	9 (0–80)	
BM diagnosed by		
CT	341	74.3
MRT	111	24.2
Investigation leading to diagnosis unknown	7	1.53
Detection of primary tumor		
Chest X-ray	**101**	**22.0**
CT chest/abdomen	193	42.0
FDG PET	15	3.3
Dermatological investigation	4	0.9
Mammography	3	0.7
Endoscopy	8	1.7
Mediastinoscopy	4	0.9
Bronchoscopy	14	3.1
Ultrasound	4	0.9
Gynecological examination	2	0.4
BM histology	33	7.2
Autopsy	10	2.2
BM biopsy or surgery performed		
Yes	302	65.8
No	157	34.2
Primary tumor tissue analyzed		
Yes	212	46.2
Primary tumor diagnosis confirmed	159	34.6
Primary tumor not diagnosed	53	11.5
No	247	53.8

Significant *p* values are marked in bold

Distinct diagnosis of a primary tumor type could be made in 374/459 (81.5%) patients within 3 months after BM diagnosis. In 250/374 (66.8%) patients, the primary tumor could be identified within the first 2 weeks after BM diagnosis. However, identification of the primary tumor required > 14 days in 71/374 (19.0%) patients and > 30 days in 53/374 (14.2%) patients. Median latency from diagnosis of BM to diagnosis of the primary tumor was 9 days (range 0–80). In 10/459 (2.2%) patients post mortem autopsy revealed primary tumor diagnosis. In 1/459 (0.2%) patient examined with post mortem autopsy, no extracranial primary tumor as cause of BM could be identified.

The most frequently identified primary tumor was lung cancer in 308/459 (67.1%) patients, followed by melanoma in 26/459 (5.7%), renal cell carcinoma in 21/459 (4.6%), colorectal carcinoma in 9/459 (2.0%), other rare tumor entities in 6/459 (1.3%) patients and breast cancer in 4/459 (0.9%) patients. In 4/26 (15.4%) melanoma patients the primary tumor lesion could be located whereas in 22/26 (84.6%) patients no extracranial melanoma lesion was located and therefore the primary tumor was defined as occult melanoma.

In 3/459 (0.7%) patients two extracranial tumor types were identified simultaneously within 30 days after BM diagnosis. In 1/459 (0.2%) patient with histologically proven BM from colorectal carcinoma, a locally advanced colorectal carcinoma and a locally restricted melanoma were detected during diagnostic workup. In 1/459 (0.2%) patient histological workup of BM tissue revealed the diagnosis of metastatic lung cancer. The further workup resulted in the diagnosis of locally advanced lung cancer and locally restricted breast carcinoma. In 1/459 (0.2%) patient extracranial biopsy as well as BM biopsy revealed the diagnosis of metastatic lung cancer. However, in post mortem autopsy a small intestine tumor was detected additionally (see Supplementary Table 2).

Interestingly, 6/459 (1.3%) patients developed an additional extracranial malignancy during the further course of disease (1/459 (0.2%) lung cancer, 1/459 (0.2%) breast cancer, 2/459 (0.4%) renal cell carcinoma, 1/459 (0.2%) squamous cell skin cancer, 1/459 (0.2%) gastric autonomic nerve tumor) (see Supplementary Table 3).

### Clinical characteristics of CUP BM patients

**Fig. 2:**
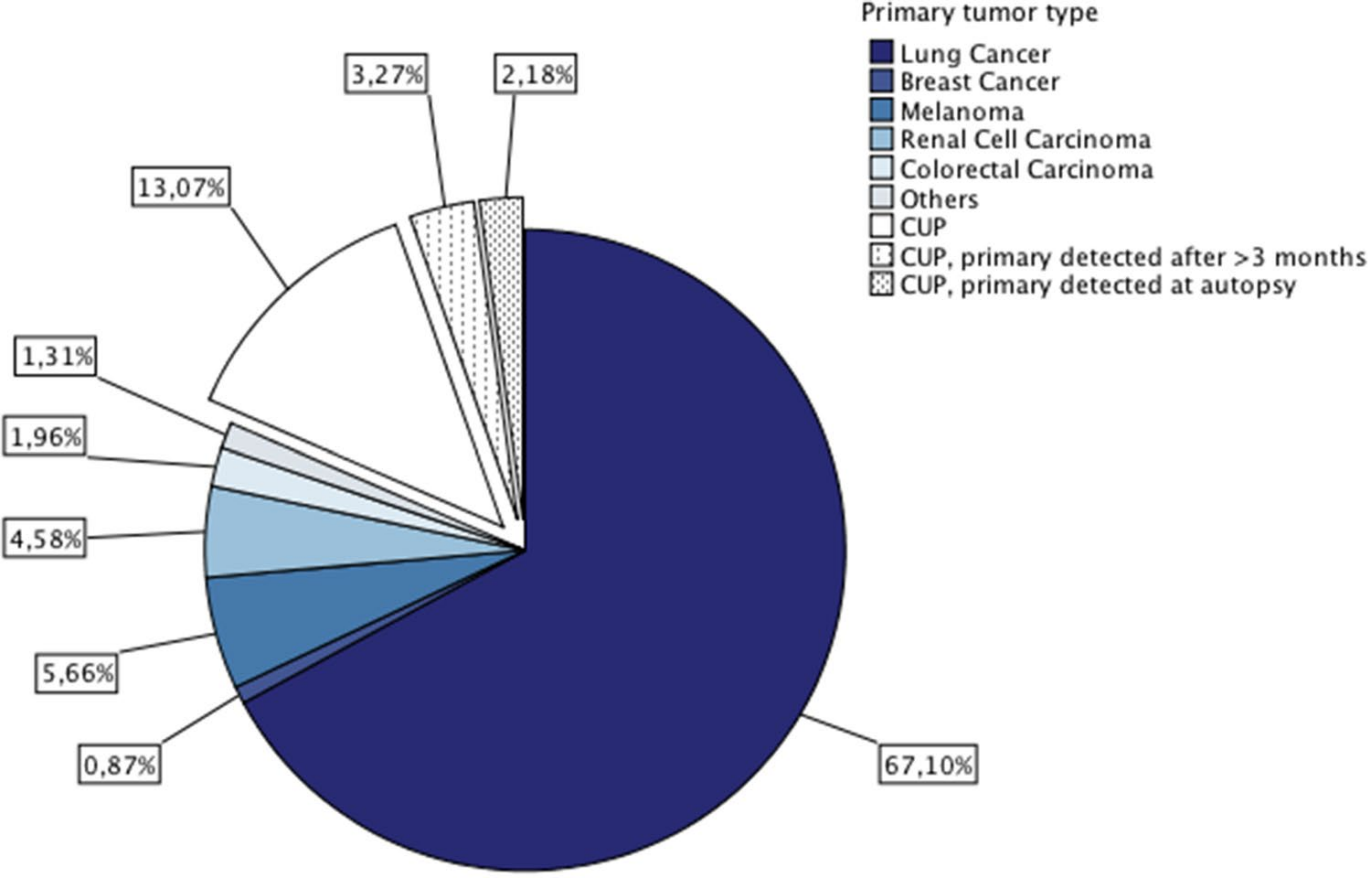
Distribution of primary tumor types

**Fig. 3:**
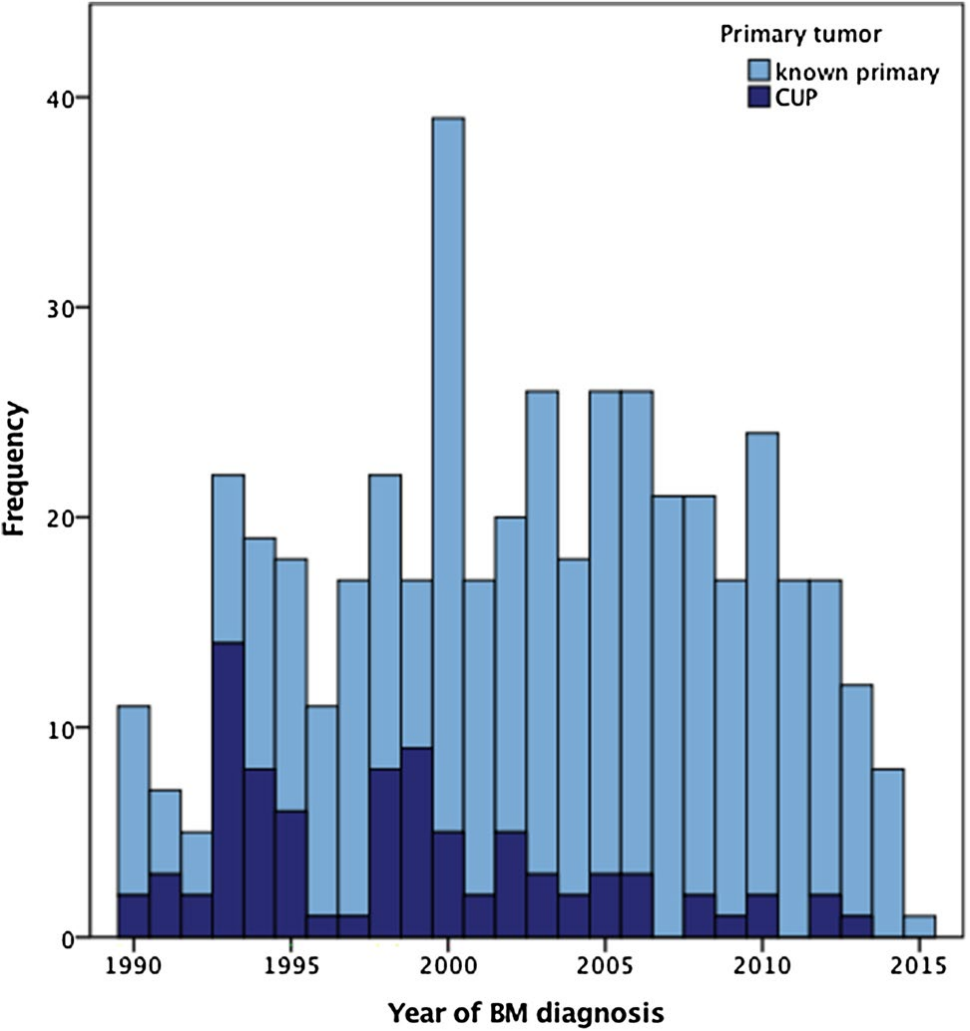
Amount of patients with identified primary tumor and CUP plotted over time

In 85/459 (18.5%) patients (36/85 (42.4%) female, 49/85 (57.6%) male), no primary tumor could be identified within 3 months after BM diagnosis despite intensive diagnostic workup and these patients were defined as patients with cancer of unknown primary (CUP) in the further analysis (See Figs. 1, 2). The rate of CUP BM patients was statistically higher in the time period 1990–1999 compared to 2000–2015 (54/149 (36.2%) vs. 31/310 (10%); p < 0.001; Chi Square test; Fig. 3).

Clinical characteristics in CUP patients and patients with BM as first symptom of cancer but identified primary tumor within 3 months after initial presentation were comparable regarding gender (p = 0.878; Chi Square test), age at BM diagnosis (p = 0.081; Chi Square test), distribution of GPA classes (p = 0.253; Chi Square test) and number of BM (p = 0.809; Chi Square test; Table 3). Presence of extracranial tumorous lesions was less common in CUP patients than in patients with identified primary tumor (13/85 (15.3%) vs. 163/374 (43.6%); p < 0.001; Chi Square test). Importantly, extracranial tumorous lesions were a- to oligosymptomatic as the leading symptom causing the diagnostic work up was the neurological symptoms caused by BM. Initial presentation with a KPS < 70 was more common in the group of CUP patients compared to patients with known primary tumor (21/85 (24.7%) vs. 57/374 (15.2%); p = 0.036; Chi square test).

**Table 3:** Comparison of clinical characteristics between brain metastases patients with cancer of unknown primary and identified primary tumor

Parameter	CUP (n = 85)		Known primary (n = 374)		p-value
	n	%	n	%	
Age at BM diagnosis years (range)	61 (29–86)		59 (27–88)		0.628
Gender					
Female	36	42.4	155	41.4	0.878
Male	49	57.6	219	58.6	
KPS at BM diagnosis (range)	70 (10–100)		80 (10–100)		0.082
> 70	64	75.3	317	84.8	**0.036**
< 70	21	24.7	57	15.2	
Number of BM					
1	39	45.9	177	47.3	0.809
2–3	27	31.8	106	28.3	
> 3	19	22.4	91	24.3	
Extracranial tumorous lesions					
Yes	13	15.3	163	43.6	< **0.001**
No	72	84.7	211	56.4	
Pulmonary tumorous lesions					
Yes	7	8.2	74	19.8	**0.012**
No	78	91.8	300	80.2	
Liver tumorous lesions					
Yes	8	9.4	29	7.8	0.612
No	77	90.6	345	92.2	
Symptomatic burden					
Oligo-to asymptomatic	1	1.2	3	0.8	0.737
Symptomatic	84	98.8	371	99.2	
First-line BM					
Gamma Knife	7	8.3	87	23.3	< ** 0.001**
Chemotherapy	1	1.2	1	0.3	
Surgery	59	70.2	216	57.8	
WBRT	11	13.1	68	18.2	
Best supportive	6	7.1	2	0.5	
Chemotherapy after diagnosis of BM					
Yes	25	29.4	197	52.7	< ** 0.001**
No	60	70.6	177	47.3	
Targeted therapy after diagnosis of BM					
Yes	2	2.4	27	7.2	0.096
No	83	97.6	347	92.8	
Extracranial progression after 1st line therapy					
Yes	29	34.1	177	47.3	**0.027**
No	56	65.9	197	52.7	
Intracranial progression after 1st line therapy					
Yes	29	34.1	157	42.0	0.183
No	56	65.9	217	58.0	

Significant *p* values are marked in bold

CUP BM patients were more likely to be treated by neurosurgical resection (60/85 (70.6%) vs. 216/374 (57.8%); p = 0.029; Chi Square test) while treatment with stereotactic radiosurgery (SRS) was more common in patients with identified extracranial primary tumor (7/85 (8.2%) vs. 87/374 (23.3%); p = 0.002; Chi Square test). CUP BM patients were less likely to receive chemotherapy during their clinical course as compared to patients with identified extracranial primary tumor (25/85 (29.4%) vs. 197/374 (52.7%); p < 0.001; Chi Square test).

Interestingly, subsequent radiological investigation could reveal an extracranial primary tumor diagnosis after a time period of > 3 months in 15/85 (17.6%) patients. In these patients, initial treatment decisions already had to be made prior to identification of the underlying primary tumor. In 9/85 (10.5%) of these CUP BM patients, diagnosis of an extracranial malignancy was possible 3–6 months after BM diagnosis. In 5/85 (5.8%) of these CUP BM patients the extracranial malignancy was identified 6–12 months after the diagnosis of BM and in 1/85 (1.8%) patient the diagnosis of the extracranial malignancy was possible only after 1 year following the diagnosis of BM. Supplementary table 4 compares clinical factors between CUP BM patients without diagnosis of a primary tumor during their life time and BM patients with diagnosis of a primary tumor during their course of disease.

### Survival analysis

#### Survival analysis in patients with BM as first manifestation of advanced cancer

Median OS among patients with BM as first manifestation of cancer was 8 months (range 0–230). GPA class presented with significant association with survival prognosis. Patients categorized in GPA class I (51/459; 11.1% patients) presented with a median OS of 17 months, compared to 14 months in class II (61/459; 13.3% patients), 7 months in class III (276/459; 60.1% patients) and 4 months in class IV (71/459; 15.5% patients; p < 0.001; log rank test) (See Table 4).

**Table 4:** Survival analysis in patients with brain metastases as first symptom of advanced cancer

Characteristic	Median OS (months)	p-Value log rank test
GPA		
Class I	17	< **0.001**
Class II	14	
Class III	7	
Class IV	4	
Primary tumor type		
Lung cancer	8	0.818
Breast cancer	3	
Melanoma	6	
Renal cell carcinoma	9	
Colorectal carcinoma	9	
CUP	5	
Others	5	
Primary tumor		
Identified primary tumor	8	0.417
CUP	5	
First-line treatment		
Surgery	9	< ** 0.001**
Gamma Knife	6	
WBRT	5	
Best supportive care	1	
Chemotherapy	< 1	
Targeted therapy		
Received	20	**0.003**
Not received	7	

Significant *p* values are marked in bold

**Fig. 4:**
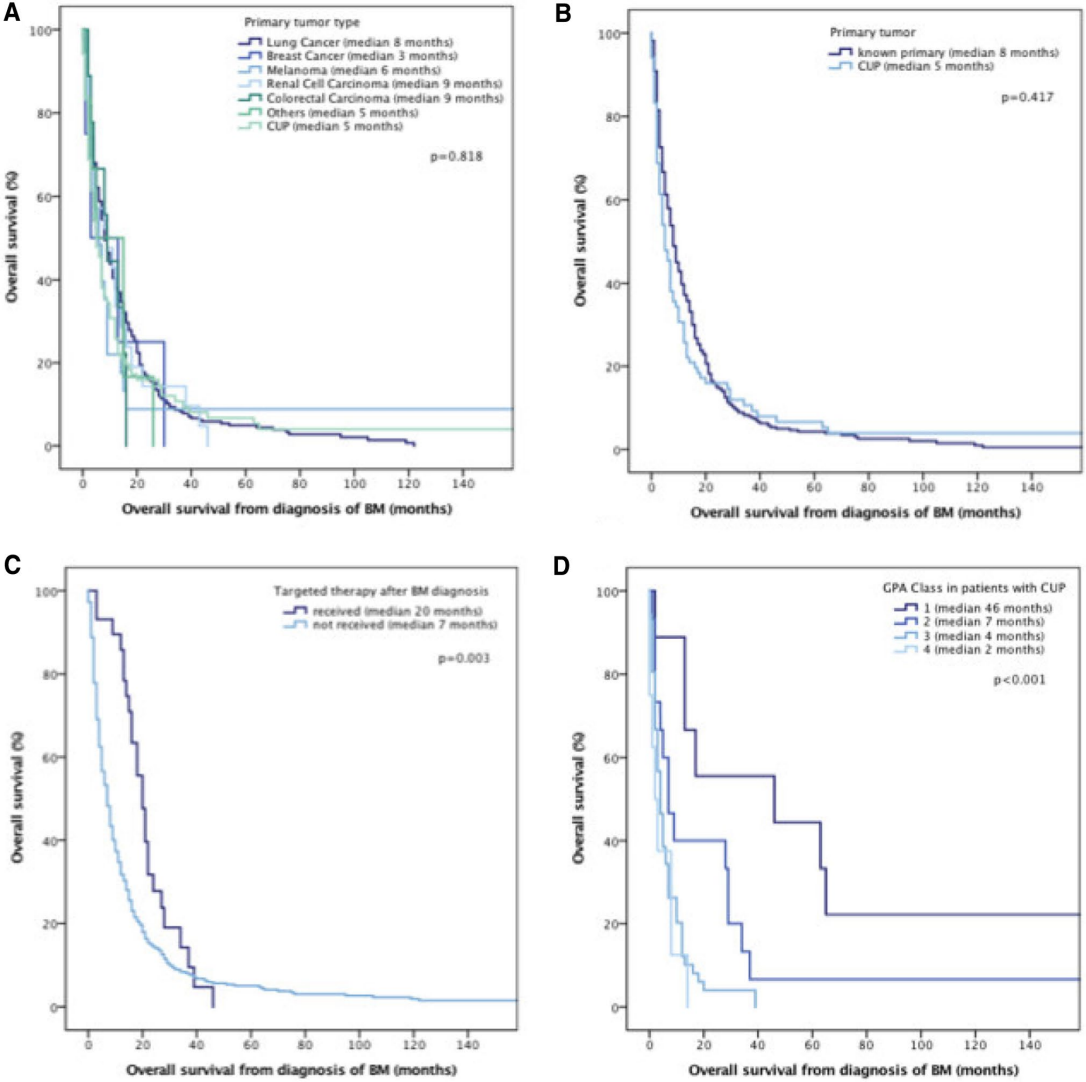
Overall survival (**a**) according to primary tumor type (**b**) in comparison between CUP and known primary (**c**) according to administration of targeted therapy (**d**) in the subgroup of CUP patients according to GPA class

The type of primary tumor was not associated with survival prognosis. Longest survival was observed in renal cell and colorectal carcinoma (9 months), followed by lung cancer (8 months), melanoma (6 months), rare tumor entities and CUP (5 months) and breast cancer (3 months) (p = 0.818; log rank test; Fig. 4a). Patients with occult melanoma showed no impaired survival compared to patients with known primary melanoma lesion (6 months vs. 6 months; p = 0.468; log rank test). Latency from BM diagnosis to primary tumor diagnosis did not show significant influence on survival prognosis (p = 0.520; Cox-regression model with time dependent variable). Further, patients with CUP (defined as no definitive tumor diagnosis within 3 months after diagnosis) did not present with a statically significant different survival prognosis as compared to patients with known extracranial primary tumor (5 months vs. 8 months; p = 0.417, log rank test; Fig. 4b). Survival in initial CUP patients with late diagnosis (> 3 months after initial presentation) of the primary tumor was longer (median 14 months) compared to CUP patients without diagnosis of a primary tumor during their life time (median 4 months) and patients with BM as first symptom of cancer and subsequent diagnosis of a primary tumor within 3 months (median 8 months; p = 0.002; log rank test).

Patients with targetable mutation as identified by molecular analysis were more likely to receive targeted therapy (26/212 (12.3%) vs. 3/247 (1.2%); p < 0.001; Chi Square test) (See Table 3). The application of targeted therapy presented with a statistically significant longer OS of 20 months as compared to 7 months in patients without (p = 0.003; log rank test; Fig. 4c).

#### Prognostic parameters in CUP BM patients

CUP BM patients presented with a median OS of 5 month and GPA class presented with statistical significant association with OS prognosis. Patients in GPA class I (9/85; 10.6%) presented with a median OS of 46 months, patients in class II (15/85; 17.6%) with 7 months, class III (53/85; 62.4%) with 4 months and class IV (8/85; 9.4%) with 2 months (p < 0.001; log rank test; Fig. 4d).

## Discussion

Patients with BM as first manifestation of advanced cancer remain a clinical challenge in oncology. Multimodal diagnostic assessment and therapy planning need to be coordinated, oftentimes in patients with acute neurological symptoms that require adequate care. Exact knowledge about primary tumor distribution and prognostic factors is important to guide diagnostic assessment and treatment planning [1]. Here we were able to investigate prognostic factors in patients with BM as first manifestation of advanced cancer in a large real-life cohort from a tertiary care center.

Lung cancer was the most frequently identified site of origin in the present cohort of patients with BM as first symptom of advanced cancer, although the frequency was with 67.1% slightly higher than the 43% reported in the overall BM population [1, 8]. In line, lung cancer patients have a particular risk for simultaneous diagnosis of BM and primary tumor compared to other extracranial tumor entities. Thus, our findings support results from previous studies indicating that 60–72% lung cancer patients but only 2–4% of breast cancer and 3–10% of melanoma patients present with simultaneous diagnosis of BM and primary tumor [1–3, 9, 10]. Indeed, localization in the lung itself might promote brain metastatic spread as lung metastases have been postulated as a risk factor for the development of BM in extracranial malignancies [11, 12].

No primary tumor could be identified within 3 months after initial presentation in 18.5% of patients with BM as first symptom of advanced cancer. This represents a considerable smaller fraction than in previous studies postulating that no primary tumor can be identified in 25–37% of BM patients [1–3, 13–15]. As these studies investigated patients in the time period before 2000, this difference may be accounted for by improvement in diagnostic process as well as implementation of FDG-PET in extended diagnostic assessment in recent years [16]. Splitting up our cohort in patients with BM diagnosis before and after 2000, we observed a significantly smaller amount of only 10% remaining with the diagnosis CUP in the latter group, supporting this hypothesis. Indeed, a recently published series, including BM patients with unknown primary diagnosed between 2004 and 2014, suggested that primary tumor detection as well as prognostic assessment was improved by adding FDG-PET to the diagnostic work flow [16]. Of note, in 17.6% of CUP patients the primary tumor could finally be identified after > 3 months during subsequent checkup and staging. These patients presented with significantly enhanced survival estimation compared to other CUP patients, probably due to both the benefits from late systemic manifestation and the later applicability of tumor adapted chemotherapy. As in two-thirds of these cases the diagnosis was made by CT or PET-CT scan, it appears that continuation of diagnostic follow-up using these techniques may be beneficial even if initial investigations were inconclusive [16].

Interestingly, we observed a high frequency of melanoma patients with occult primary lesion (84.6%). This represents a considerable higher amount compared to previous studies that report 1–6% of melanoma being from occult primary [17–20]. The difference may be due to the particularities of our cohort that excludes patients with systemic tumor manifestation, favoring a high prevalence of patients with non-detectable primary melanoma lesion. According to a meta-analysis by Bae et al., patients with occult metastatic melanoma show better survival than those with detected primary tumor [20]. However, in our cohort no difference in survival prognosis between patients with detected primary melanoma lesion and occult melanoma could be observed.

Patients with identified primary tumor within 3 months after initial presentation did not show better survival than those with CUP, being in line with previous investigations [21–25]. No statistical difference was observed despite the frequent absence of extracranial disease in CUP patients, although absence of extracranial disease is as a favorable prognostic parameter in several established prognostic assessments including the GPA score [5]. Therefore, the brain metastatic disease might determine survival in a subgroup of patients, as underscored by the observation that approximately one-third of unselected BM patients die from intracranial progression in the absence of extracranial progression [8]. Some primary tumors, however, show a more uneven distribution as almost half of breast cancer and colorectal cancer patients present with intracranial progression and stable extracranial disease in the last 8 weeks before cancer related death, indicating that intracranial tumor control is the life limiting factor in these patients [8]. Molecular factors potentially causing the brain specific spread of the observed BM CUP patients might also facilitate the life limiting brain progress. Specific genetic alterations like activating mutations in the cyclin dependent kinase (CDK) or mechanistic Target of Rapamycin (mTOR) pathway were identified in the BM but absent in the matched primary tumor, indicating that these specific mutations might be associated with BM progression and therefore emerging targets for BM specific therapies [26].

Patients with BM as the first symptom of advanced cancer, subsequent diagnosis of a primary tumor and molecular workup were more likely to receive targeted therapies. In line, patients treated with a targeted therapy after diagnosis of BM in the present cohort of patients with BM as first symptom of advanced cancer presented with improved survival compared to patients without the possibility of a targeted therapy approach. This finding further underscores the importance of molecular workup as administration of personalized treatment approach was shown to improve outcomes in the hard-to-treat cancer population [27, 28]. We could show that the GPA score, a commonly used score for prognostic assessment of BM patients and an important basis for clinical decision making, is also of prognostic value in the specific cohort of patients presenting with BM as first manifestation of cancer and the sub-cohort of CUP BM patients. Our data may be useful for planning of emerging clinical trial efforts that use advanced molecular diagnostics to enroll CUP patients into clinical trials using targeted therapies, as the GPA may help to select or stratify patients for such trials.

Patients with BM as first symptom of cancer presented more frequently with symptoms of increased intracranial pressure, neurological deficits or epileptic seizures compared to patients with known primary tumor disease and subsequent diagnosis of BM. This finding might be explained by the higher percentage of patients with known primary tumor diagnosed in a- to oligosymptomatic stadium, as already mild neurological symptoms indicate additional cranial imaging [29]. Further, patients with known metastatic lung cancer or melanoma are frequently staged for intracranial involvement, although clinical practice concerning the systematic staging for BM is heterogenous trough Europe [30, 31]. Subsequently patients identified through screening are also more likely to have a- to oligosymptomatic disease, causing the difference in symptomatic intensity. Among patients with BM as first symptom of advanced cancer, no difference in the BM related symptom intensity was observed between patients with subsequently identified primary tumor and CUP patients. The BM related symptomatic burden was the leading clinical sign in all patients of our cohort. The different pattern in the symptomatic burden of these cancer patients is also addressed in the treatment approach, as immediate local therapies are needed in patients with highly symptomatic BM while a systemic treatment approach utilizing therapies with high intracranial efficacy can be considered in selected a- to oligosymptomatic BM patients [29]. Therefore, our findings underscore the clinical heterogeneity of BM patients, as well as the importance for individual clinical decision making in a multidisciplinary team.

The findings of our study are certainly limited by the disadvantages of its retrospective design. As patients with extracranial symptoms and detection of BM during the staging process were excluded in order to accomplish a homogenous cohort of patients with BM as the first clinical sign of cancer, the prevalence of patients with undetectable primary tumor might be overestimated. Since our study investigated a long-time period including different eras of imaging technology, modern cohorts might differ slightly due to easier access to modern imaging techniques including FDG-PET. Nevertheless, we were able to investigate a comprehensive set of clinical data in a large single center cohort of patients with BM as the first symptom of metastatic cancer and provide important information on prognostic factors and clinical characteristics within this special cohort.

In conclusion, we were able to investigate a large unique real-life cohort of patients with BM as first manifestation of advanced cancer. The acquired knowledge of this study revealed that even if initial investigations fail to reveal the underlying primary tumor entity, diagnostic assessment should be continued as some patients experience late diagnosis. Molecular workup of the primary tumor should be conducted whenever possible as tumor-specific targeted therapies may enhance survival times. Due to improvement of diagnostic assessment and implementation of personalized therapy approaches in recent years, a reasonable survival even in this clinically challenging cohort of CUP BM patients is possible.

## Electronic supplementary material

Below is the link to the electronic supplementary material.

Supplementary material 1 (DOCX 21 KB)

## Abbreviations

BM: Brain metastases
CUP: Cancer of unknown primary
GPA: Graded prognostic assessment
KPS: Karnofsky performance score
MRT: Magnetic resonance tomography
CT: Computer tomography
OS: Overall survival
WBRT: Whole brain radiotherapy
CDK: Cyclin dependent kinase
mTOR: Mechanistic Target of Rapamycin
