# Supplementary material for: Brain metastases as first manifestation of advanced cancer: exploratory analysis of 459 patients at a tertiary care center
Source: Clin Exp Metastasis. 2018 Nov 12;35(8):727–38. doi: 10.1007/s10585-018-9947-1 (PMC6267666; doi:10.1007/s10585-018-9947-1)
Supplement: Supplementary file 1 — Supplementary material 1 (DOCX 21 KB) [file 10585_2018_9947_MOESM1_ESM.docx]

**Supplementary Material**

**Supplementary Table 1.** Characteristics of patients with BM and a second primary tumor before BM diagnosis (n=20)

| **Primary tumor type of BM** | **BM origin identified by** | **Localized**  **tumor treated with curative intend prior to BM** | **Time between diagnosis of localized**  **tumor treated with curative intend and BM diagnosis** |
| --- | --- | --- | --- |
| Lung cancer | BM histology | Prostate cancer | 8 years |
| Lung cancer | BM histology | Bladder cancer | 10 years |
| Lung cancer | BM histology | Melanoma | 3 years |
| Lung cancer | BM and lymph node histology | Cervical cancer | 20 years |
| Lung cancer | BM histology | Vaginal cancer | 12 years |
| Lung cancer | BM histology | Prostate cancer | 7 years |
| Lung cancer | BM histology | Melanoma | 2 years |
| Lung cancer | BM histology | Renal cell carcinoma | 6 years |
| Lung cancer | BM histology | Cervical cancer | 4 years |
| Lung cancer | BM and bone metastasis histology | Melanoma | 17 years |
| Lung cancer | Histology of peripheral metastasis | Cervical cancer | 12 years |
| Lung cancer | Peripheral lymph node histology | Cervical cancer | 28 years |
| Lung cancer | Cured early-stage skin cancer | Squamous cell skin cancer | 7 years |
| Lung cancer | Cured localized bladder cancer | Bladder cancer | 20 years |
| Lung cancer | Surgically cured early-stage melanoma | Melanoma | 3 years |
| Lung cancer | BM histology | Breast cancer | 7 years |
| Melanoma | Histology of lung metastasis | Breast cancer | 4 years |
| Melanoma | Histology of liver metastasis | Colorectal carcinoma | 27 years |
| Melanoma | BM histology | Non-Hodgkin-lymphoma  Prostate cancer  Renal cell carcinoma | 11 years  6 years  5 years |
| Lung cancer | Surgically cured localized prostate cancer | Prostate cancer | 1 year |

**Supplementary Table 2.** Characteristics of patients with BM and two primary tumors simultaneously diagnosed with BM (within 30 days after BM diagnosis) (n=3)

| **Primary tumor type of BM** | **BM origin identified by** | **Second primary tumor simultaneously diagnosed with BM (within 30 days)** | **Time between diagnosis of BM and second primary tumor** |
| --- | --- | --- | --- |
| Colorectal carcinoma | Histology of BM and extracranial metastasis | Melanoma | < 30 days |
| Lung cancer | Localized breast cancer | Breast cancer | < 30 days |
| Lung cancer | BM histology | Colorectal carcinoma | < 30 days, at autopsy |

**Supplementary Table 3.** Characteristics of patients with BM and a second primary tumor diagnosed during clinical course after BM diagnosis (n=6)

| **Primary tumor type of BM** | **BM origin identified by** | **Second primary tumor diagnosed during clinical course after BM diagnosis** | **Time between diagnosis of BM and second primary tumor diagnosed during clinical course** |
| --- | --- | --- | --- |
| CUP | BM histology (squamous cell carcinoma) not concordant with renal cell carcinoma | Renal cell carcinoma | 3 years |
| CUP | BM histology (adenocarcinoma) not concordant with GAN tumor | Gastric autonomic nerve tumor (GAN tumor) | 2 years |
| Lung cancer | BM histology | Squamous cell skin cancer | 6 months |
| Lung cancer | BM histology | Renal cell carcinoma | 7 months |
| Lung cancer | BM histology | Breast cancer | 2 months |
| Melanoma | BM histology | Lung cancer | 15 years |

**Supplementary Table 4** Comparison of clinical characteristics between brain metastases patients with cancer of unknown primary during the life time, patients with late diagnosis of the primary tumor (> 3 months after initial presentation) and patients with BM as first symptom of advances cancer and identified primary tumor within 3 months after initial presentation

| **Parameter** | **CUP (n=70)** | | **Late diagnosis (> 3 months; n=15)** | | **Known primary (n=389)** | | **p-value** |
| --- | --- | --- | --- | --- | --- | --- | --- |
|  | **n** | **%** |  | | **n** | **%** |  |
| Age at BM diagnosis years (range) | 62  (29-86) | | 59  (45-70) | | 59  (27-88) | | 0.632 |
| Gender |  |  |  |  |  |  | 0.968 |
| Female | 30 | 42.9 | 6 | 40.0 | 155 | 41.4 |  |
| Male | 40 | 57.1 | 9 | 60.0 | 219 | 58.6 |  |
| KPS at BM diagnosis  (range) | 70  (10-100) | | 80  (60-100) | | 80  (10-100) | | **0.003** |
| >70 | 51 | 72.9 | 13 | 86.7 | 317 | 84.8 | **0.048** |
| <70 | 19 | 27.1 | 2 | 13.3 | 57 | 15.2 |  |
| Number of BM |  |  |  |  |  |  | 0.975 |
| 1 | 32 | 45.7 | 7 | 46.7 | 177 | 47.3 |  |
| 2–3 | 22 | 31.4 | 5 | 33.3 | 106 | 28.3 |  |
| >3 | 16 | 22.9 | 3 | 20.0 | 91 | 24.3 |  |
| Extracranial tumorous lesions |  |  |  |  |  |  | **<0.001** |
| Yes | 12 | 17.1 | 1 | 6.7 | 163 | 43.6 |  |
| No | 58 | 82.9 | 14 | 93.3 | 211 | 56.4 |  |
| First-line BM |  |  |  |  |  |  | **<0.001** |
| Gamma Knife | 5 | 7.1 | 2 | 14.3 | 87 | 23.3 |  |
| Chemotherapy | 1 | 1.4 | 0 | 0.0 | 1 | 0.3 |  |
| Surgery | 48 | 68.6 | 11 | 78.6 | 216 | 57.8 |  |
| WBRT | 11 | 15.7 | 0 | 0.0 | 68 | 18.2 |  |
| Best supportive | 5 | 7.1 | 1 | 7.1 | 2 | 0.5 |  |
| Chemotherapy after diagnosis of BM |  |  |  |  |  |  | **<0.001** |
| Yes | 14 | 20.0 | 11 | 73.3 | 297 | 52.7 |  |
| No | 56 | 80.0 | 4 | 26.7 | 177 | 47.3 |  |
| Targeted therapy after diagnosis of BM |  |  |  |  |  |  | 0.188 |
| Yes | 1 | 1.4 | 1 | 6.7 | 27 | 7.2 |  |
| No | 69 | 98.6 | 14 | 93.3 | 347 | 92.8 |  |
| Extracranial progression after 1^st^ line therapy |  |  |  |  |  |  | **0.007** |
| Yes | 20 | 28.6 | 9 | 60.0 | 177 | 47.3 |  |
| No | 50 | 71.4 | 6 | 40.0 | 297 | 52.7 |  |
| Intracranial progression after 1^st^ line therapy |  |  |  |  |  |  | **<0.001** |
| Yes | 17 | 24.3 | 12 | 80.0 | 157 | 42.0 |  |
| No | 53 | 75.7 | 3 | 20.0 | 217 | 58.0 |  |
| Detection of primary tumor |  |  |  |  |  |  | **<0.001** |
| Chest X Ray | 0 | 0.0 | 1 | 7.7 | 100 | 27.2 |  |
| CT Chest/Abdomen | 0 | 0.0 | 6 | 46.2 | 187 | 50.8 |  |
| FDG PET | 0 | 0.0 | 4 | 30.8 | 11 | 3.0 |  |
| Dermatological investigation | 0 | 0.0 | 0 | 0.0 | 4 | 1.1 |  |
| Mammography | 0 | 0.0 | 0 | 0.0 | 3 | 0.8 |  |
| Endoscopy | 0 | 0.0 | 0 | 0.0 | 8 | 2.2 |  |
| Mediastinoscopy | 0 | 0.0 | 0 | 0.0 | 4 | 1.1 |  |
| Bronchoscopy | 0 | 0.0 | 0 | 0.0 | 12 | 3.3 |  |
| Ultrasound | 0 | 0.0 | 0 | 0.0 | 4 | 1.1 |  |
| Gynecological examination | 0 | 0.0 | 0 | 0.0 | 2 | 0.5 |  |
| BM histology | 0 | 0.0 | 0 | 0.0 | 33 | 9.0 |  |
| Autopsy | 10 | 24.3 | 0 | 0.0 | 0 | 0.0 |  |
| Overall survival from diagnosis of BM, months  (range) | 4  (0-230) | | 14  (5-196) | | 8  (0-178) | | **0.002** |
